# Anti-Mold Effectiveness of a Green Emulsion Based on *Citrus aurantium* Hydrolate and *Cinnamomum zeylanicum* Essential Oil for the Modern Paintings Restoration

**DOI:** 10.3390/microorganisms10020205

**Published:** 2022-01-19

**Authors:** Maura Di Vito, Lara Vergari, Melinda Mariotti, Maria Rita Proto, Lorenzo Barbanti, Stefania Garzoli, Maurizio Sanguinetti, Luigia Sabatini, Alice Peduzzi, Maria Grazia Bellardi, Paola Mattarelli, Francesca Bugli, Daphne De Luca

**Affiliations:** 1 Dipartimento di Scienze Biotecnologiche di Base, Cliniche Intensivologiche e Perioperatorie, Università Cattolica del Sacro Cuore, Largo A. Gemelli 8, 00168 Rome, Italy; melinda.mariotti@unicatt.it (M.M.); maurizio.sanguinetti@unicatt.it (M.S.); francesca.bugli@unicatt.it (F.B.); 2 Department of Pure and Applied Sciences (DiSPeA), University of Urbino Carlo Bo, Piazza della Repubblica, 13, 61029 Urbino, Italy; laravergari@gmail.com (L.V.); daphne.deluca@uniurb.it (D.D.L.); 3 Department of Agricultural and Food Sciences, University of Bologna, Viale G. Fanin 42, 40127 Bologna, Italy; mariarita.proto2@unibo.it (M.R.P.); lorenzo.barbanti@unibo.it (L.B.); mariagrazia.bellardi@unibo.it (M.G.B.); paola.mattarelli@unibo.it (P.M.); 4 Dipartimento di Chimica e Tecnologie del Farmaco, Università di Roma Sapienza, Piazzale Aldo Moro 5, 00185 Rome, Italy; stefania.garzoli@uniroma1.it; 5 Dipartimento di Scienze di Laboratorio e Infettivologiche, Fondazione Policlinico Universitario A. Gemelli IRCCS, Largo A. Gemelli 8, 00168 Rome, Italy; 6 Dipartimento di Scienze Biomolecolari, Sezione di Farmacologia e Igiene, Università degli Studi di Urbino Carlo Bo, 61029 Urbino, Italy; luigia.sabatini@uniurb.it; 7 Dipartimento di Biologia Ambientale, Sapienza Università di Roma, Piazzale Aldo Moro 5, 00185 Rome, Italy; alice.peduzzi@gmail.com

**Keywords:** essential oil, hydrolate, modern painting, cultural heritage, mold

## Abstract

A modern painting is characterized by multi-material bases extremely exposed to biodeteriogenic attacks. The aim of this work was to test the antifungal effectiveness of a natural, eco-friendly, and safe emulsion based on *Citrus aurantium* L. var. *amara* hydrolate and *Cinnamomum zeylanicum* Blume (from bark) essential oil, named “*Zeylantium green* emulsion” (Zege), on modern paintings. Colored unaged and aged canvas samples, performed with modern techniques (acrylic, vinylic and alkyd), were used to test in vitro both the antifungal effectiveness of Zege and its impact on the chemical–physical characteristics. Microbiological tests were performed according to the EUCAST international guidelines. pH measurements and colorimetric analysis were performed on unaged and aged canvases before and after Zege spray treatment. Finally, in situ tests were performed using the spray emulsion on canvas samples obtained from Ilaria Margutti’s modern artwork, which had been colonized by molds. Microbiological tests on canvas prototypes showed a time- and dose-dependent effectiveness of the Zege spray. None of the techniques underwent relevant changes in pH. Only the acrylic colors were unaffected in the colorimetric analysis, among all colored unaged or aged canvases. Tests made with modern artwork samples confirmed the in situ antifungal effectiveness. The Zege spray showed encouraging results in regard to the use of this formulation in the restoration of modern paintings.

## 1. Introduction

Modern art, an initial form of rejection of past currents, fills a long chapter of history that began at the end of the Nineteenth Century and represents the desire for experimentation towards new techniques and visual forms. It developed through a vast series of artistic currents, often also very different from each other involving illustrious names from Romanticism to Futurism, Cubism and Surrealism, and contemporary art, still in constant evolution, in the search for new materials, forms, and subjects.

A modern painting is characterized by material bases that give a three-dimensional shape to the picture using different effects: striped, spatulated, sponged, etc. This polymaterial nature makes these paintings extremely exposed to biodeteriogenic attacks, which can irreversibly damage this heritage of humanity. In addition to poly-materials, it is possible to distinguish two types of paints, solvent-based paint and water-based paint. Water-based paints are the most susceptible to attack by microorganisms that survive in this element [1].

Biodeteriogens (bacteria, archaea, fungi, lichens, and insects) are a serious danger for the conservation of all cultural heritage including that made with modern materials. Climate, conservation, the chemistry, and the composition of the materials define the rate of microbial colonization and are at the basis of biodeterioration phenomena. Therefore, one of the main challenges in managing biodeterioration is the control of microbial growth to avoid the complete destruction of the canvas painting. The decontamination of these works requires the use of chemicals with a high toxic potential for both humans and the environment, besides high costs for museums [2,3].

Several measures are planned to delay, control, or stop the presence of biodeteriogens (especially molds) on cultural heritage materials. Disinfection practices are performed before any other manipulation to avoid the spreading of fungal spores potentially present on artworks, which may be dangerous for both the artworks and human health. Chemical formulations such as Biotin R, Biotin T, and Preventol^®^ RI80/RI50 are often used by restorers to prevent or treat fungal colonization. Biotin R is composed by iodiopropynyl butylcarbamate (IPCB) and n-octyl-isothiazolinone (OIT), dissolved in 2(2-butoxy-ethoxy), while Biotin T by OIT and a quaternary ammonium salt. The first is generally used at 3% in ligroin, petroleum essence, or white spirit, while the second one at 3% in demineralized water, ethyl alcohol, or butyl acetate. Preventol^®^ RI80/RI50 is a concentrated formulation with a broad spectrum characterized by quaternary ammonium salts generally diluted from 2–5% in water or ethanol [4,5,6].

Although these are the main products in use for the restoration of paintings, contemporary art paintings present many critical issues as they are characterized by substances that are potentially sensitive to most of the solvents used (including water when used without changing their conductivity and pH).

Furthermore, it is highlighted that sensitization in addition to mutagenicity (ability to induce genetic mutations), carcinogenicity (ability to induce cancer), teratogenicity (ability to induce anomalies in embryonic development), and embryotoxicity (ability to induce toxic effects on the embryo) are the main toxic effects attributed to chemicals generally used in formulations for artwork restoration [7,8,9].

Problems related to both the restoration practices of modern artworks and human health have resulted in the study of green restoration products by several groups, who have focused their attention on the effectiveness of various natural substances. Among these, essential oils (EOs) and hydrolates (Hys), both obtained through the distillation of vegetable parts of aromatic plants, are raising much interest in Italy and abroad. Both EOs and Hys are natural products with antimicrobial activity, but they differ because the former have a high anti-microbial activity and are hydrophobic natural phytocomplexes so concentrated that they cannot be used a pure because of potentially toxicity. The latter are natural hydrophilic products, with a lower anti-microbial activity due to the extreme dilution of their active components, but they are more versatile, safe, and inexpensive. This last characteristic depends on Hys being by-products of the distillation process aimed for EO extraction from various plant organs.

Hys have only recently been discovered in the cultural heritage environment because their characteristics make them less aggressive than EOs and applicable in contexts where the latter cannot be applied such as the restoration of paper works. In this context, the fungicidal activity of *C. aurantium* var. *amara* Hy was exploited in combination with the gel of gellan to clean paper artworks and at the same time inactivate the fungal biodeteriogens [10].

The purpose of this work was to test both in vitro and in situ the anti-fungal effectiveness of an emulsion based on *Citrus aurantium* L. var. *amara* Hy and *Cinnamomum zeylanicum* Blume (from bark) EO in order to identify new cytocidal formulations useful in the decontamination of modern paintings, which should be economical, but most importantly have a low environmental impact and high safety for human health.

## 2. Materials and Methods

### 2.1. Canvas Samples

Table 1 shows the three types of techniques used to perform canvases in order to imitate modern paintings. For in vitro microbiological test, the first type of samples was used, consisting of 51 small canvas squares (SCSs) of 2 × 2 cm, performed with modern techniques. For in situ experiments, the second type of samples was used, consisting of two large canvas samples simulating modern painting techniques carried out as described in Table 1. The specimen support was a rough canvas with a cotton flap and industrial preparation. The canvas was then stretched on a custom-made wooden frame (26 cm high × 18 cm wide × 2.5 cm thick).

### 2.2. Headspace-Gas Chromatography and Mass Spectroscopy Analysis

The chemical analyses of vapor and liquid phase *C. zeylanicum* EO were conducted by a headspace connected to the gas chromatograph coupled with a mass spectrometer (Clarus 500—Perkin Elmer, Waltham, MA, USA). A Varian Factor Four VF-1 capillary column was used and helium as the gas carrier at a flow of 1 mL/min. The temperature program of oven GC was as follows: 60 °C for 5 min and ramped to 220 °C at a rate of 5 °C/min for 20 min. The ionization energy of MS was 70 eV and the scan range 40–450 *m*/*z*. The compounds were identified by the comparison of mass spectra with those of authentic standards from the Wiley and NIST libraries. Furthermore, the linear retention indices (LRIs) were calculated with reference to the series of n-alkanes (C8–C30 aliphatic hydrocarbons, Ultrasci, Bologna, Italy). For the quantification of the identified compounds, GC-FID analysis was performed following the same conditions reported above. Relative percentages were obtained by peak area normalization, and no internal standard or correction factors were used. The analysis was repeated in triplicate.

### 2.3. Spray Formulation

In this work, the emulsion named “*Zeylantium green* emulsion” (Zege) was previously tested in vitro to evaluate its antimicrobial effectiveness on canvas samples made according to modern techniques and infected with a fungal suspension specified in Section 2.4. (data not shown). The Zege was composed by 0.03% *v*/*v* of the *Cinnamomum zeylanicum* EO from bark (Pranarom International, Ghislenghien, Belgium) in 99.97% *v*/*v* of *Citrus aurantium* var. *amara* Hy from flowers (Erboristeria Magentina s.r.l., Turin, Italy).

### 2.4. In Situ Microbiological Test

The in situ fungicidal effectiveness of the spray formulation (see Section 2.3) was assessed using the SCSs. All canvases were previously sterilized by UV light for 80 min (40 min per side). A 50 μL fungal suspension characterized by fungi mainly associated with biodeteriogenic attack of artworks (2.5 × 10^5^ CFU/mL, equal amount of *Aspergillus niger* (ATCC9642), *Aureobasidium pullulans* (ATCC 15233), *Chaetomium globosum* (ATCC 6205), *Cladosporium cladosporioides* (ATCC 16022), *Alternaria alternata,* and *Penicillium citrinum*) was inoculated on the back of the canvases. Once dried, 1 to 5 sprays (capacity of 55 μL/spray i.e., 14 μL/cm^2^ of each SCS) of the formulation were applied on the back of SCSs for 3 h, 5 h, or 24 h. The treated side of the canvas was sown on PDA (Oxoid, Basingstoke, UK) and incubated for 7 d at 30 °C. Treatment efficacy was evaluated at the end of the incubation. Untreated and uninoculated canvas (positive and negative control, respectively) were included in the tests as well. Every treatment was performed in triplicate.

### 2.5. Chemical–Physical Analysis

Aged and unaged samples were chemically and physically analyzed (see the next sections) pre- and post-anti-microbial treatment, to evaluate if the application of the spray mixture (Section 2.3) could modify the chemical–physical or structural properties of the canvas, preparatory layers, and/or paint film.

#### 2.5.1. Ageing Conditions

“Solar box 3000e RH Xenon” (provided with a xenon lamp, outdoor filter (280 nm), and radiation power of 550 W/m^2^) was used to age BCSs for 3 wk in order to assess the treatment efficacy progression in time. The aging process was achieved with hot/humid conditioning cycles at 30 °C and relative humidity between 55% and 99% for 72 h, interposed with 55% atmospheric humidity cycles for 96 d. During the months in which the specimens aging took place, the cycle was endlessly reiterated.

#### 2.5.2. Spray Treatment

The BCSs were cautiously released from the support, positioned in a glass container with the paint layer heading down; a Melinex^®^ sheet separated the glass and paint surfaces. An amount of 50 sprays (14 μL/cm^2^) of freshly prepared mix (see Section 2.3) was employed for the treatment: 25 sprays were applied to the back of the canvas samples and 25 on a sheet of acid-free absorbent paper placed in direct contact with the back of the canvas. To prevent the movement of the textile fibers and to better maintain both surfaces in contact with one another, five weights were laid on the upper part of the absorbent paper. In the end, the glass container was secured with a special lid to avoid the evaporation of the volatile components and incubated at 30 °C for 5 h. The treatment was repeated in triplicate.

#### 2.5.3. pH Measurements

The pH measurement was based on the Cremonesi (2012) technique [4]: 3 discs (3 mm of diameter) of 2% (*w*/*v*) agar (AGAR AGAR—ANTARES, Bologna, Italy) were set on the surface of each sample for 3 min. The disc was placed in direct contact with the sensor, and the pH was evaluated using the “ISFET Mini-Lab^®^” model. The pH was measured before and after natural treatment on both aged and unaged canvases, and the pH difference between before and after treatment was calculated for each color, painting technique, and ageing condition. Data were submitted to a two-tailed *t*-test to assess statistical differences determined by treatment The CoStat 6.3 package (Cohort, Monterey, CA, USA) was used to perform the *t*-test.

#### 2.5.4. Colorimetric Measurements

The “Konica Minolta CM-2600d” (with diffuse illumination, 8° display with simultaneous measurement of specular component included (SCI) and specular component excluded (SCE) with γ rays, λ 360–740 nm) was used for the colorimetric measurements. Colorimetric data are reported as a function of standard CIE D65 illumination and 10° (CIE 1964) supplementary standard observer excluding the specular component of radiation. The colorimetric coordinates L*, a*, b* were determined as a function of the CIEL*a*b 1976 color space [10]. Differences in color (ΔE), chroma (ΔC), and brightness (ΔL), together with the mean (M) and standard deviation (SD), were calculated starting from the L*, a*, b* values according to the 2000 CIE guidelines [11]. All measurements were performed in triplicate. Every Δ value was interpreted as follows: 0–1 (chromatic difference not detectable by the human eye), 1–3 (small chromatic difference), 3–6 (detectable difference), or >6 (large difference). The values greater than 3 were the only ones evaluated as significant since the values ≤ 3 were imperceptible or considered irrelevant.

### 2.6. Treatment of the Artwork

**Figure 1:**
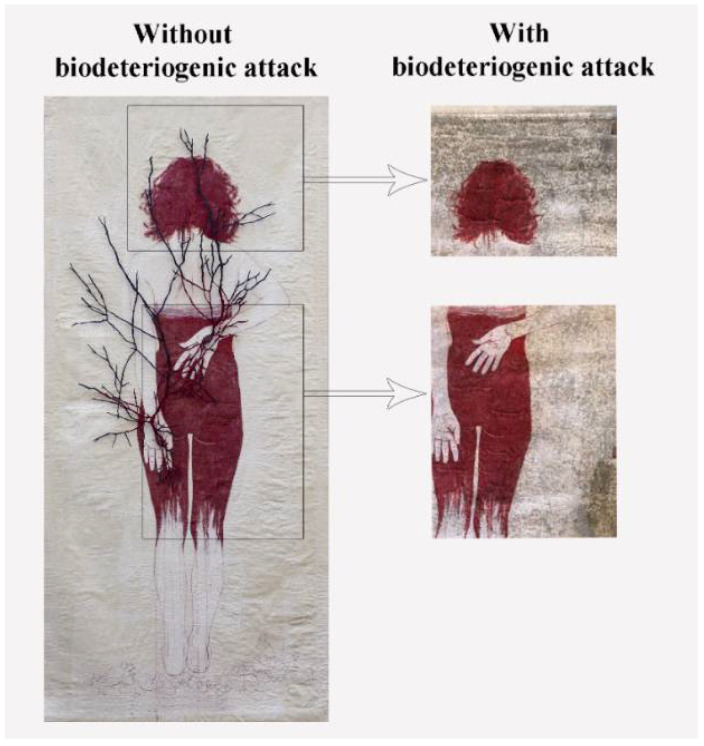
“Du sang, de la Mort et de la Volupté” by Ilaria Margutti. On the left is the painting before the biodeteriogenic attack. On the right, the details of the painting after the biodeteriogenic attack.

The contemporary fungal-colonized painting named “Du sang, de la Mort et de la Volupté” by Ilaria Margutti (Figure 1; year: 2017, dimensions: 80 × 200 cm) exhibited during the “Volta e Rivolta” shown at the Collective Art Gallery, Galleria Elettra (Sant’Anna del Furlo, Pesaro Urbino, Italy) was used as a case study after the authorization of the artist. Painting canvas samples were treated with the Zege formulation in order to verify its antifungal activity.

#### 2.6.1. Sampling and Microbial Analysis of the Artwork

Before the spray treatment, three cotton swabs (Boettger, Paul Boettger GmbH & Co., KG, Bodenmais, Germany) and three fungi-tapes (Scientific Device, Glenview, IL, USA) were collected on the painting to identify the microbial biodeteriogens. Samples were sown on the following nutrient agars: Mueller Hinton agar, malt extract agar, and PDA (all by Oxoid, Basingstoke, UK) and incubated at 30 °C for 7 d. After the incubation, the identification of the fungal strains was performed using matrix-assisted laser desorption ionization time-of-flight mass spectrometry (MALDI-TOF MS) (Bruker Daltonics, Bruker, Bremen, Germany).

#### 2.6.2. Spray Cytocidal Treatment

To verify the anti-fungal action of the spray mixture, 6 samples of canvas of 2 × 2 cm with an evident macroscopic contamination were taken: 3 were treated with the spray mixture, while the remaining 3 were not treated (pre-treatment controls). The treated ones were placed in a sterile container, and 4 sprays were applied (capacity of 130 µL/spray). The container was sealed with laboratory film (Parafilm^®^, Bemis Company, Inc., Neenah, WI, USA) to avoid the evaporation of the mixture components and incubated at 30 °C for 5 h. Later, both the treated and the untreated samples were seeded on PDA (Oxoid, Basingstoke, UK) nutrient agar and incubated at 30 °C for 7 d.

## 3. Results

### 3.1. Gas Chromatography and Mass Spectroscopy Analysis 

A total of twenty-four compounds were detected and listed in Table 2. Oxygenated monoterpenes were the most abundant components both in the liquid phase and in the vapor phase (9.6% and 34.7%, respectively). Cinnamaldehyde was the major compound of the *C. zeylanicum* EO (66.0% liquid phase and 24.5% vapor phase) followed by *β*-caryophyllene (5.8%; 19.2%), linalool (4.9%; 17.2%), 1,8-cineole (4.4%; 17.5%), and *p*-cymene (3.8%; 8.2%). (*E*)-Cinnamyl acetate was detected only in the liquid phase (5.5%), while *α*-thujene reached an important level only in the vapor phase (8.3%).

### 3.2. In Situ Microbiological Test

Table 3 shows data from the in situ tests conducted with SCS. The Zege spray showed a time- and dose-dependent effectiveness. Three sprays were active after 24 h of incubation, while five sprays are effective already after 2 h of incubation.

### 3.3. Ageing Conditions

The modern techniques were highly resistant to the aging process. Techniques present on BCSs exhibited only some mechanical stress caused by the cotton canvas, which is particularly sensitive to water.

### 3.4. pH Measurements

pH data are showed in Table 4. Variations of the pH after treatment were less than 0.5 units of pH. There were both increases and decreases, which were significant at *p* < 0.5 in four cases out of twenty-four.

### 3.5. Colorimetric Measurements

Table 5 shows the DE values obtained after the treatment of aged and unaged canvases. Most of the detections (twenty out of twenty-four) showed values of the color difference (∆E) lower than three, while only four conditions showed significant changes. Specifically, after treatment, vinyl blue showed significant variation in both unaged and aged samples, while blue and red alkyd showed variations only in unaged samples. Red and green colors did not show variations in any of the three techniques.

### 3.6. Sampling and Microbial Analysis of the Artwork

Microbiological analysis revealed that the artwork was contaminated by *Rhizopus stolonifer*, *Penicillium* spp., *Alternaria alternata*, and *Aspergillus* spp. The major area of the painting was colonized by *R. stolonifer*.

### 3.7. Spray Cytocidal Treatment

**Figure 2:**
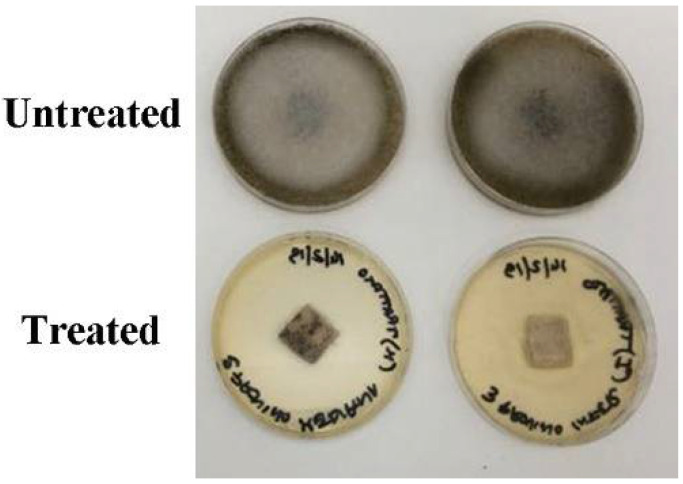
Canvases taken from the artwork of contemporary art. “Untreated” canvases had no kind of treatment. “Treated” canvas were treated with three sprays of the Zege emulsion and incubated 24 h.

Figure 2 shows the cytocidal effectiveness of the treatment. The Zege was applied as described in Section 2.6.2; at the end of the incubation time, all treated samples were free of fungal growth.

## 4. Discussion

Due to both the nature of the binders and the different products and materials, the conservation and restoration of paintings of contemporary art require a different approach than ancient artworks. Acrylic, alkyd, and vinyl binders, as well as young oil are substances potentially sensitive to most of the solvents used (including water). Young oil, for example, is subjected to swelling and leaching and is sensitive to polar solvents and chelators. Synthetic binders are subject to aging and wrinkling of the pictorial film, the migration of fatty acids, and other components that cause blooming, stiffening of the film, cracking, rapid yellowing and darkening (alkyd binder), photodegradation due to UV, cross-linking, deacetylation (vinyl binder), strong swelling, and hypersensitivity to polar organic solvents and water (acrylic binder) [11,12,13].

In recent years, researchers of cultural heritage have been turning their attention to green restoration, looking for new natural products that are at the same time active against biodeteriogens and safe for human health. Several research groups are studying the potential uses of EOs and, more recently, Hys (by-products of distillation) in restoration practices. As of late, both *Origanum vulgare* L. and *Thymus vulgaris* L., *Coridothymus capitatus* L., *Syzigium aromaticum* L., and *Cinnamomum zeylanicum* Blume EOs have been in situ studied against biodeteriogens of stone artworks [14,15,16,17]. Thymol and eugenol (major components of *T. vulgaris* and *O. vulgare* EOs) have shown interesting results, in the restoration of wall paintings [18]. In ex situ studies, *O. vulgare* EO was tested on biodeteriogenic fungal strains isolated from the Serbian monastery Holy Virgin Church [14]. Borrego et al. tested *S. aromaticum*, *Allium sativum* L., and *O. vulgare* EOs against biodeteriogens isolated from the National Archive of the Republic of Cuba and the Historical Archive of the Museum of La Plata, Argentina [19]. Other ex situ studies have tested the efficacy of *Cinnamomum camphora* J. Presl, *Helichrysum italicum* Roth, *Lavandula angustifolia* Miller, and *Cymbopogon winterianus* Jowitt ex Bor EOs against fungal biodeteriogens [14,16,17,18,19,20]. *C. zeylanicum* EO has been poorly studied in restoration. In particular, two types of applications were tested: the first was through the nebulization of this EO to preserve ancient textile fibers, while the second exploited the effectiveness of the EO encapsulated in psyllium-alginate beads [21,22]. Considering the antimicrobial efficacy already established in the literature of *C. zeylanicum* EO and its vapors, we decided to study the activity of this EO in association with the *C. aurantium* var *amara* Hy already known as an anti-fungal treatment for cultural heritage [10].

Our in vitro results relating to the cytocidal activity of the OE (MFC_average_ = 0.5% *v*/*v*) and of the Hy (MFC_average_ = 2.6% *v*/*v*) and the data obtained in this study confirmed the in vitro and in situ antimicrobial activity of Zege against fungal biodeteriogenic strains.

In vitro experiments indicated that a concentration of 28 μL/cm^2^ of Zege was effective on modern art canvases already 5 h after treatment. After Zege application, both unaged and aged canvases did not show significant pH variations. The only notable exceptions are both acrylic ocher and vinylic green unaged samples, which however showed only very slight variations. Significant variations on aged canvases (vinyl red and alkyd blue) are not of great practical interest as the modern artworks that require restoration are, at the moment, unaged. Instead, the colorimetric measurements showed that only the acrylic colors do not undergo significant variations after the treatment. Some vinyl and alkyd colors may undergo important variations. Therefore, further research is needed for these types of colors. 

Although the mechanisms of action of *C. zeylanicum* EO have not been elucidated in the field of cultural heritage, other studies showed that the liquid or vapor phase of its major component, cinnamaldehyde, inhibits molds by inhibiting ATPases and cell wall biosynthesis and by altering the membrane structure and integrity [23]. Similarly, linalool alcohol, which is the main component of *C. aurantium* var. *amara* Hy, has shown, among other properties, an antimicrobial property by altering the cell membrane and inhibiting the formation of biofilm [24]. Therefore, it is plausible that the synergy between the EO and the Hy contained in Zege acts on fungal biodeteriogens, inhibiting the fungal biofilm present on the artwork and killing the fungal cells through the mechanisms set out.

Following the in vitro results, it was decided to take and treat samples obtained from the no-dye canvas of the painter Ilaria Margutti’s modern artwork. Tests carried out on no-dye canvases of the “Du sang, de la Mort et de la Volupté” painting confirmed the fungicidal activity of the Zege formulation by showing, 5 h after treatment, the complete killing of the fungal load present on the painting (Figure 2).

If formulated by hand, the spray mixture must be made at the moment, otherwise the small concentration of volatile terpene components decays over time with consequent loss of the cytocidal effectiveness. To optimize the activity of the Zege spray, the canvas must be isolated from the surrounding environment, creating a confined environment capable of retaining the volatile terpenic compounds in direct contact with the artwork, increasing its effectiveness. Furthermore, for at least 5 h, the artwork must be placed at a controlled temperature (about 30 °C) in order to induce the germination of spores of biodeteriogenic fungi and allowing the mixture to kill them in their weakest vital phase. Finally, it is advisable to administer the treatment on the back of the canvas and on acid-free paper sheets to be placed in contact with the back to avoid the evaporation of the product.

Moreover, Zege is safe for the health of restorers because the amount of chemicals, reported by the reference organization (IFRA) as toxic, is in line with thresholds indicated for products for human use. In particular, the concentration of safrole is enormously lower than 0.1% (0.00018% *v*/*v*), and that of cinnamaldehyde is between 0.014% and 1.8%, as required for products for human use (the Zege cinnamaldehyde content equals 0.02%).

## 5. Conclusions

Zege was studied to be a possible alternative to biocides normally used in the restoration of canvas artworks. The spray formulation showed encouraging and statistically significant results regarding the stability of the pH and color values after the treatment of contemporary artworks made with acrylic colors. In addition, it is a green formulation, safe for human health and the environment, releasing a delicate fragrance generally well accepted by the operators. However, further studies are needed to validate the effectiveness of Zege on paintings made with other modern techniques and colonized by a greater number of biodeteriogens.

## Figures and Tables

**Table 1:** Modern techniques: description of preparatory and pictorial layers.

Sample	Technique	Preparation	Paint Layer
Ac	Acrylic	Industrial	Industrial colors: bright red, ultramarine blue, green, yellow ocher
V	Vinyl	Industrial	Industrial colors: ruby red, ultramarine blue, green, yellow ocher
Al	Alkyd	Industrial	Industrial colors: cadmium red, ultramarine blue, green earth, yellow ocher

**Table 2:** Chemical composition (percent mean values) of *C. zeylanicum* EO.

N°	Component ^1^	LRI ^2^	LRI ^3^	EO (%) ^4^	EO (%) ^5^
1	*α*-thujene	920	923	0.1	8.3
2	*α*-pinene	938	943	2.7	tr
3	camphene	942	946	0.2	tr
4	*β*-pinene	985	986	0.2	tr
5	*α*-phellandrene	998	996	0.4	1.4
6	*p*-cymene	1020	1026	3.8	8.2
7	1,8-cineole	1022	1027	4.4	17.5
8	*γ*-terpinene	1050	1054	tr	tr
9	cis-linalool oxide	1060	1058	tr	tr
10	linalool	1089	1092	4.9	17.2
11	camphor	1130	1126	0.1	-
12	*α*-terpineol	1019	1021	0.1	tr
13	4-terpinenyl acetate	1283	1286	0.1	tr
14	*O*-anisaldehyde	1250	1242 *	0.1	-
15	cinnamaldheyde	1269	1275	66.0	24.5
16	eugenol	1333	1331	4.1	tr
17	*β*-caryophyllene	1429	1426	5.8	19.2
18	(*E*)-cinnamyl acetate	1441	1439	5.5	-
19	humulene	1450	1454	0.4	tr
20	eugenol acetate	1480	1483	0.1	-
21	*O*-methoxy cinnamaldheyde	1507	1505	0.3	-
22	*δ*-cadinene	1529	1530	0.1	tr
23	caryophyllene oxide	1577	1583	0.3	tr
24	benzyl benzoate	1741	1739	0.3	-
	SUM			100.0	96.3
	Monoterpene hydrocarbons			7.4	17.9
	Oxygenated monoterpenes			9.6	34.7
	Sesquiterpene hydrocarbons			6.3	19.2
	Oxygenated sesquiterpene			0.3	-
	Others			76.4	24.5

^1^ The components are reported according to their elution order on the apolar column; ^2^ linear retention indices measured on the apolar column; ^3^ linear retention indices from the literature; * LRI not available; ^4^ percent mean values of *C. zeylanicum* EO (liquid phase); ^5^ percent mean values of *C. zeylanicum* EO (vapor phase)—not detected; tr: traces (mean value < 0.1%).

**Table 3:** Effectiveness of Zege sprays according to the number of sprays and time of incubation.

N. of Spray	Hours		
	2	5	24
5	−	−	−
4	+/−	−	−
3	+	+	−
2	+	+	+

(−) Absence of growth; (+/−) growth inhibition; (+) growth.

**Table 4:** pH values of canvases before and after treatment.

Color	Technique	Unaged				Aged			
		A-BT ^1^	A-AT ^2^	AV ^3^	SD ^4^	A-BT	A-AT	AV	SD
Red	Ac ^5^	7.00	6.73	**−0.27 (n.s.)**	**0.32**	6.63	6.83	**0.20 (+)**	**0.00**
	V ^6^	6.70	6.67	**−0.03 (n.s.)**	**0.06**	6.93	6.47	−0.47 (**)	**0.06**
	Al ^7^	6.23	6.53	**0.30 (n.s.)**	**0.10**	6.67	6.30	**−0.37 (+)**	**0.12**
Blue	Ac	6.83	6.73	**−0.10 (n.s.)**	**0.10**	6.67	6.77	**0.10 (n.s.)**	**0.10**
	V	6.73	6.67	**−0.07 (n.s.)**	**0.12**	6.67	6.70	**0.03 (n.s.)**	**0.06**
	Al	6.30	6.53	**0.23 (n.s.)**	**0.15**	6.67	6.40	−0.27 (*)	**0.06**
Green	Ac	7.27	6.83	**−0.43 (+)**	**0.23**	6.87	6.93	**0.07 (n.s.)**	**0.06**
	V	6.63	6.80	0.17 (*)	**0.06**	6.80	6.70	**−0.10 (n.s.)**	**0.10**
	Al	6.17	6.27	**0.10 (n.s.)**	**0.10**	6.27	6.17	**−0.10 (n.s.)**	**0.20**
Ocher	Ac	7.03	6.70	−0.33 (*)	**0.06**	6.47	6.73	**0.27 (+)**	**0.15**
	V	6.47	6.57	**0.10 (n.s.)**	**0.10**	6.67	6.70	**0.03 (n.s.)**	**0.06**
	Al	6.43	6.40	**−0.03 (n.s.)**	**0.23**	6.50	6.40	**−0.10 (n.s.)**	**0.10**

^1^ Average before treatment; ^2^ average after treatment; ^3^ average variation; ^4^ standard deviation; ^5^ acrylic; ^6^ vinyl; ^7^ alkyd. n.s., (+), * and ** mean non-significant and significant at *p* < 0.10, 0.05 and 0.01, respectively.

**Table 5:** Mean values of the colorimetric parameters.

		Unaged Canvas (Mean Values)								Aged Canvas (Mean Values)							
		Before Treatment			After Treatment					Before Treatment			After Treatment				
**Color**	**Sample**	L*	a*	b*	L*	a*	b*	**∆E**	**SD-DE ^1^**	L*	a*	b*	L*	a*	b*	**∆E**	**SD-DE**
Red	A ^2^	48.9	57.5	46.5	49.5	58.0	46.5	1.15	0.44	47.5	55.2	40.8	46.9	54.8	40.4	0.82	0.09
	V ^3^	43.3	58.7	30.9	43.6	58.7	30.6	0.60	0.22	42.8	57.5	28.8	42.6	57.5	30.1	1.33	0.21
	Al ^4^	50.8	54.7	37.4	50.8	55.4	38.2	1.06	0.17	48.0	53.2	35.3	49.1	53.7	35.8	1.35	0.17
Blue	A	28.0	26.2	−51.9	27.2	27.1	−52.7	1.4	0.05	29.2	28.3	−56.3	27.9	29.2	−55.9	1.73	0.08
	V	29.0	34.2	−65.1	27.2	29.7	−56.1	10.3	4.64	32.6	30.2	−63.8	30.3	33.9	−65.9	4.89	0.28
	Al	26.0	15.4	−30.8	25.3	15.8	−31.2	3.73	1.70	32.2	21.6	−56.6	32.2	20.2	−54.4	2.69	0.45
Green	A	35.7	−3.33	7.79	35.8	−3.18	7.51	0.35	0.22	35.7	−2.97	8.59	34.9	−2.98	7.94	1.07	0.21
	V	37.7	−7.36	12.8	37.9	−7.35	12.5	0.44	0.07	37.7	−7.41	13.5	37.0	−7.28	13.0	1.09	0.36
	Al	31.6	−9.32	6.26	32.9	−10.8	7.39	2.28	1.00	36.5	−9.55	4.81	36.4	−9.55	5.14	1.56	0.50
Ocher	A	63.3	15.2	45.6	63.4	15.3	45.5	0.45	0.15	61.3	15.1	45.2	61.3	15.2	44.8	1.48	0.81
	V	65.5	14.5	48.6	64.6	14.6	47.9	1.15	0.20	65.4	14.5	48.1	64.4	14.8	48.5	1.60	1.04
	Al	57.7	17.3	45.8	50.1	15.4	36.3	12.5	14.3	49.8	14.9	34.4	49.3	14.7	33.8	0.82	0.50

^1^ Standard deviation of ∆E; ^2^ acrylic; ^3^ vinyl; ^4^ alkyd.

## Data Availability

Not applicable.
